# Urine metabolomics and microbiome analyses reveal the mechanism of anti-tuberculosis drug-induced liver injury, as assessed for causality using the updated RUCAM: A prospective study

**DOI:** 10.3389/fimmu.2022.1002126

**Published:** 2022-11-22

**Authors:** Ming-Gui Wang, Shou-Quan Wu, Meng-Meng Zhang, Jian-Qing He

**Affiliations:** ^1^ Department of Respiratory and Critical Care Medicine, West China Hospital, Sichuan University, Chengdu, China; ^2^ Department of Emergency Medicine, Sichuan Provincial People’s Hospital, University of Electronic Science and Technology of China, Chengdu, China

**Keywords:** metabolomic, microbiome, anti-tuberculosis drug-induced liver injury (ATB-DILI), machine learning, cohort, updated RUCAM

## Abstract

**Background:**

Anti-tuberculosis drug-induced liver injury (ATB-DILI) is one of the most common adverse reactions that brings great difficulties to the treatment of tuberculosis. Thus, early identification of individuals at risk for ATB-DILI is urgent. We conducted a prospective cohort study to analyze the urinary metabolic and microbial profiles of patients with ATB-DILI before drug administration. And machine learning method was used to perform prediction model for ATB-DILI based on metabolomics, microbiome and clinical data.

**Methods:**

A total of 74 new TB patients treated with standard first-line anti-TB treatment regimens were enrolled from West China Hospital of Sichuan University. Only patients with an updated RUCAM score of 6 or more were accepted in this study. Nontargeted metabolomics and microbiome analyses were performed on urine samples prior to anti-tuberculosis drug ingestion to screen the differential metabolites and microbes between the ATB-DILI group and the non-ATB-DILI group. Integrating electronic medical records, metabolomics, and microbiome data, four machine learning methods was used, including random forest algorithm, artificial neural network, support vector machine with the linear kernel and radial basis function kernel.

**Results:**

Of all included patients, 69 patients completed follow-up, with 16 (23.19%) patients developing ATB-DILI after antituberculosis treatment. Finally, 14 ATB-DILI patients and 30 age- and sex-matched non-ATB-DILI patients were subjected to urinary metabolomic and microbiome analysis. A total of 28 major differential metabolites were screened out, involving bile secretion, nicotinate and nicotinamide metabolism, tryptophan metabolism, ABC transporters, etc. Negativicoccus and Actinotignum were upregulated in the ATB-DILI group. Multivariate analysis also showed significant metabolic and microbial differences between the non-ATB-DILI and severe ATB-DILI groups. Finally, the four models showed high accuracy in predicting ATB-DILI, with the area under the curve of more than 0.85 for the training set and 1 for the validation set.

**Conclusion:**

This study characterized the metabolic and microbial profile of ATB-DILI risk individuals before drug ingestion for the first time. Metabolomic and microbiome characteristics in patient urine before anti-tuberculosis drug ingestion may predict the risk of liver injury after ingesting anti-tuberculosis drugs. Machine learning algorithms provides a new way to predict the occurrence of ATB-DILI among tuberculosis patients.

## Introduction

Tuberculosis (TB) is caused by *Mycobacterium tuberculosis* infection, which is an infectious disease with the highest mortality before the novel coronavirus pneumonia pandemic (1). According to the report of the World Health Organization, there were 9.9 million new cases of tuberculosis worldwide, and approximately 1.3 million patients died in 2020 (1). After treatment with a first-line anti-tuberculosis regimen containing isoniazid and rifampicin, 86% of patients were treated successfully (1–3). However, it is often accompanied by various adverse drug reactions, such as gastrointestinal reaction, drug-induced liver injury (DILI), hyperuricemia, leucopenia, allergy, peripheral neuritis and so on (3–7). Anti-tuberculosis drug-induced liver injury (ATB-DILI) is one of the most common adverse reactions in the treatment of tuberculosis (4–6) and may lead to treatment interruption, prolonged treatment time, decreased treatment success rate and increased hospitalization rat (8–10). Early identification and evaluation of ATB-DILI will provide new ideas for the precise treatment of tuberculosis patients.

Currently, the identification of biomarkers by metabolomics has been widely used in pathophysiological mechanisms in many scientific fields, such as plant biology (11), toxicology (12) and disease diagnosis and prognosis (13–16). Ultrahigh performance liquid chromatography tandem mass spectrometry (UPLC‒MS) is one of the most effective means of metabolomics research (17). Through metabolomics research, Xie et al. found that 31 metabolites were related to DILI and were closely related to the severity of DILI (18). Prospective studies show that there are significant differences in serum metabolites between the DILI group and the non-DILI group prior to polygonum multiflorum ingestion, and the unique metabolic characteristics may be used to predict the risk of DILI after taking polygonum multiflorum (19). This suggests that metabolomics can be used to evaluate and predict DILI. There are a few clinical and animal experiments using metabolomics to explore the toxic mechanism and biomarkers of ATB-DILI (20–25). Nontargeted metabolomics found that 28 metabolites can be used as important distinguishing factors between ATB-DILI and non-ATB-DILI patients, and ATB-DILI affects the tricarboxylic acid cycle, arginine and proline metabolism, purine metabolism and pentose phosphate pathway (24). In our previous study, 11 urine differential metabolites were identified between ATB-DILI patients and non-ATB-DILI patients by gas chromatography-mass spectrometry (GC‒MS) (26). These studies indicate that metabolomics is helpful for a new understanding of the pathophysiological process of ATB-DILI and for screening new markers of ATB-DILI. Moreover, no study has evaluated the metabolic characteristics of ATB-DILI patients and non-ATB-DILI patients before taking anti-tuberculosis drugs.

There are thousands of microbial species in the human microbial ecosystem that play a key role in maintaining host immunity, metabolism, drug metabolism, vitamin production and carbohydrate metabolism (27–29). Research interest has been focused on the interaction between the microbiota and the host, and how the composition of the human microbiota may have a potential impact on the development of certain diseases, such as metabolic syndrome, obesity (30), diabetes (31) and liver injury (26, 32, 33). Previous studies have shown that the quantities of the urine microbiota differ significantly between patients with ATB-DILI and without ATB-DILI (26).

At present, the data on metabolomic or microbiota changes related to ATB-DILI are limited, especially premedication data. In addition, a model for the prediction of ATB-DILI is lacking. In this study, we hypothesized that the metabolome and microbiome are related to ATB-DILI. Therefore, we performed urine metabolomic and microbiota analyses of ATB-DILI prior to medication. Meanwhile, four machine learning methods was used to establish a clinical prediction model of ATB-DILI based on metabolomics, microbiome and clinical data.

## Methods

### Study population and sample collection

This prospective cohort study included patients with tuberculosis who visited the tuberculosis clinic of West China Hospital of Sichuan University from March 2021 to December 2021. The study was approved by the Ethics Committee of West China Hospital of Sichuan University. All research subjects were required to sign a written informed consent form by themselves or their representatives before being included in the study. Demographic datasets of patients with laboratory test data were obtained through electronic medical records and questionnaires.

Inclusion criteria are as follows: 1) age≥16 years and <80 years old; 2) newly diagnosed TB patients, including etiologically confirmed, pathologically confirmed and clinically diagnosed cases; 3) standard first-line anti-TB treatment regimens (including 2-month HRZE intensive treatment and at least 4 months of HRE consolidation therapy), and can be followed up regularly; 4) Han nationality in Southwest China; 5) voluntarily participate in this study and sign the informed consent form when included in the study. Those who do not meet the above diagnostic criteria are referred to as non-ATB-DILI.

The exclusion criteria were as follows: 1) abnormal liver function at baseline; 2) concomitant liver diseases such as alcoholic hepatitis, viral hepatitis or liver cirrhosis; 3) taking immunosuppressive drugs, antitumor drugs, and acetaminophen and other drugs that may cause liver damage; 4) patients with diabetes, autoimmune diseases, malignant tumors, or tuberculosis with severe heart, lung, and renal insufficiency; and 5) patients with HIV infection or who died during follow-up from causes other than adverse drug reactions.

The diagnostic criteria for ATB-DILI used in this study are (9, 34–36) as follows: alanine aminotransferase (ALT)≥3 normal upper limit of normal value (ULN) and/or total bilirubin (TBil)≥ 2ULN; or aspartate aminotransferase (AST) or alkaline phosphatase (ALP) and TBil are elevated at the same time, and at least one of them is ≥2 ULN. In addition, considering that the updated Roussel Uclaf Causality Assessment Method (RUCAM) was the recognized standard for the diagnosis of DILI, no matter what drug was used (37). Here, for patients who met the DILI diagnostic criteria (ALT≥5ULN or ALP≥2ULN) in the updated RUCAM, we conducted a subgroup analysis. All patients diagnosed with ATB-DILI completed a causality assessment using the updated RUCAM scale (37). Only patients with a RUCAM score of 6 or more were accepted in this study.

Urine nontargeted metabolomics and microbiome analyses were performed in ATB-DILI and gender and age-matched non-DILI patients, and patients with urinary system diseases were excluded. Urine samples were self-collected from the subjects according to the provided instructions prior to antituberculosis treatment and immediately sent to the laboratory. Once in the laboratory, samples were stored at −80°C until metabolomic and microbiome analysis.

### Sample preparation

Urine is the common sample type used to perform metabolomics studies (38, 39). Compared to other samples, urine has easy sampling, low protein levels and less complexity (38). Also the urine metabolites are products of normal and abnormal cellular biological processes and can reflect a wide range of phenotypes including genetic modifications (38). Therefore, urine is more advantageous compared to other sample types and was used as a study sample in this study.

Clean midstream urine from patients before medication was collected, divided into three 1 ml aliquots, and immediately stored at −80°C. We discarded samples that were at room temperature for >2 hours.

Metabolite extraction was primarily performed according to previously reported methods (40, 41). In short, 100 µL samples were extracted by directly adding 300 µL of precooled methanol and acetonitrile (2:1, v/v), and internal standards mix (contains: L-Leucine-d3, L-Phenylalanine (13C9, 99%), L-Tryptophan-d5, Progesterone-2,3,4-13C3) were added for quality control of sample preparation. After vortexing for 1 min and incubating at -20°C for 2 h, the samples were centrifuged for 20 min at 4000 rpm, and the supernatant was then transferred for vacuum freeze drying. The metabolites were resuspended in 150 µL of 50% methanol and centrifuged for 30 min at 4000 rpm, and the supernatants were transferred to autosampler vials for LC‒MS analysis. A quality control (QC) sample was prepared by pooling the same volume of each sample to evaluate the reproducibility of the whole LC‒MS analysis.

### Metabolite detection and comments

This experiment used a Waters 2D UPLC (Waters, USA) tandem Q Exactive HF high resolution mass spectrometer (Thermo Fisher Scientific, USA) for separation and detection of metabolites. To provide more reliable experimental results during instrument testing, the samples are randomly ordered to reduce system errors. A QC sample was interspersed for every 10 samples.

The samples were analyzed on a Waters 2D UPLC (Waters, USA), coupled to a Q-Exactive mass spectrometer (Thermo Fisher Scientific, USA) with a heated electrospray ionization (HESI) source and controlled by the Xcalibur 2.3 software program (Thermo Fisher Scientific, Waltham, MA, USA). Chromatographic separation was performed on a Waters ACQUITY UPLC BEH C18 column (1.7 μm, 2.1 mm × 100 mm, Waters, USA), and the column temperature was maintained at 45°C. The mobile phase consisted of 0.1% formic acid (A) and acetonitrile (B) in the positive mode, and in the negative mode, the mobile phase consisted of 10 mM ammonium formate (A) and acetonitrile (B). The gradient conditions were as follows: 0-1 min, 2% B; 1-9 min, 2%-98% B; 9-12 min, 98% B; 12-12.1 min, 98% B to 2% B; and 12.1-15 min, 2% B. The flow rate was 0.35 mL/min and the injection volume was 5 μL.

The mass spectrometric settings for positive/negative ionization modes (ESI+/-) were as follows: spray voltage, 3.8/−3.2 kV; sheath gas flow rate, 40 arbitrary units (arb); aux gas flow rate, 10 arb; aux gas heater temperature, 350°C; capillary temperature, 320°C. The full scan range was 70–1050 m/z with a resolution of 70000, and the automatic gain control (AGC) target for MS acquisitions was set to 3e6 with a maximum ion injection time of 100 ms. The top 3 precursors were selected for subsequent MSMS fragmentation with a maximum ion injection time of 50 ms and resolution of 30,000, and the AGC was 1e5. The stepped normalized collision energy was set to 20, 40 and 60 eV.

### LC‒MS/MS analysis

The original data (raw file) collected byLC‒MS/MS were imported into Compound Discoverer 3.1 (Thermo Fisher Scientific, USA) for data processing, including peak extraction, retention time correction, background peak labeling, and metabolite identification. We calculate the coefficient of variation of the relative peak area in all QC samples, and delete the compounds with coefficient of variation greater than 30%. The identification of metabolites was a combined result of the BGI Metabolome Database (BMDB), mzCloud and ChemSpider (Human Metabolome Database (HMDB), Kyoto Encyclopedia of Genes and Genomes (KEGG), LipidMaps) databases. Main parameters of metabolite identification: Precursor Mass Tolerance <5 ppm, Fragment Mass Tolerance <10 ppm, RT Tolerance <0.2 min. The identification level of metabolites was divided into five confidence levels, and the credibility of Level 1 to Level 5 decreased in order. The original data exported by LipidSearch were imported into metaX for data preprocessing and subsequent analysis (42). Multivariate statistical analysis (principal component analysis (PCA) and partial least squares-discriminant analysis (PLS-DA)), and univariate analysis (fold-change, FC and Student’s t test) were combined to screen for differential metabolites between groups. Differential metabolite screening conditions: 1) variable projected importance (VIP) ≥ 1, 2) fold-change ≥ 1.2 or ≤ 0.83, 3) p-value <0.05. Metabolic pathway enrichment analysis of differential metabolites was performed based on the KEGG database.

### Urine DNA extraction and 16S sequencing

Microbial genomic DNA extraction was performed as described previously (43). Urine microbial DNA was extracted using a Qiagen Mini Kit (Qiagen, Hilden, Germany) following the manufacturer’s instructions. Primers targeting the hypervariable V3+V4 region of the 16S gene were used to amplify the extracted DNA samples (the forward primer was 5ʹ- ACTCCTACGGGAGGCAGCA -3ʹ, and the reverse primer was 5ʹ- GGACTACHVGGGTWTCTAAT -3ʹ). All samples were sequenced *via* Illumina HiSeq 2500.

### Sequencing data analysis

Cutadapt v2.6 software was used to process the raw data to obtain fragments of the target region. FLASH (Fast Length Adjustment of Short reads, v1.2.11) was used for sequence splicing, and UCHIME (v4.2.40) software was used to remove chimeras. Sequences were clustered with a 97% similarity level by using USEARCH (v7.0.1090_i86linux32) to cluster the spliced tags into OTUs. The OTU representative sequences were aligned with the database for species annotation by RDP classifier (1.9.1) software, and the confidence threshold was set to 0.6. The VennDiagram package of R (v3.1.1) software was used to display the number of common and unique OTUs for each group. Principal coordinate analysis was performed using QIIME (v1.80) software to present similarities or differences in data. Line Discriminant Analysis (LDA) Effect Size (LEFSE) was used to calculate the differences in species abundance between the two groups and then to research the biomarkers related to ATB-DILI.

### Statistical analysis

Differences between two groups were compared by using Student’s t test for normal continuous variables and χ2-test for categorical variables. Differences with a p value <0.05 (two-sided) were considered statistically significant. Statistical analyses were performed using SPSS V.21.0 for Windows (SPSS, Chicago, Illinois, USA). Moreover, correlations between the microbiota and metabolites and between the metabolites and clinical parameters were analyzed. Integrating electronic medical records, metabolomic and microbiome data, and machine learning methods was used to establish a clinical prediction model of ATLI. We used four machine learning algorithms: random forest, artificial neural network, support vector machine (SVM) with the linear kernel (SVM-linear), and SVM with radial basis function kernel (SVM-rbf) (44). The stratified sampling method was used to divide the training set (80%) and the validation set (20%), and the R4.1.2 software (R Foundation for Statistical Computing, Vienna, Austria) was used for data screening and model building. The importance of each feature in the occurrence of ATB-DILI was scored, and area under receiver operating characteristic (ROC) curves were employed to assess the accuracy of the models.

## Results

### Baseline characteristics

A total of 74 patients diagnosed with TB were recruited for this study from March 2021 to December 2021 at West China Hospital of Sichuan University (Sichuan Province, China). Finally, 5 patients were lost to follow-up. Of the remaining 69 patients, 16 (23.19%) developed ATB-DILI after antituberculosis treatment (**Supplementary Figure 1**). The general clinical characteristics of the two groups and the results of liver function tests when DILI occurred are shown in **Table 1**. Compared with the non-ATB-DILI group, the levels of albumin (43.2(40.1-44.4)g/L vs. 44.9(42.2-46.8)g/L, *P*: 0.033) and hemoglobin (125.5(118.5-135.8)g/L vs. 137.0(128.5-145.5)g/L, *P*: 0.019) were significantly lower in the ATB-DILI group. No significant differences were observed in other baseline characteristics between the two groups of patients (*P*>0.05). The median time to DILI occurred on day 29 after taking anti-TB drugs (**Table 1**).

**Table 1:** Clinical characteristics of 69 tuberculosis patients.

Characteristic	ATB-DILI (n=16)	Non-ATB-DILI (n=53)	*P*
Age, years, median(IQR)	39.0(24.0-53.8)	33.0(27.0-52.0)	0.717
Females, n(%)	11(68.75)	30(56.60)	0.386
Weight, kg, median(IQR)	51.0(50.0-55.0)	55.0(49.0-60.0)	0.289
BMI, kg/m2, median(IQR)	19.9(18.9-20.9)	20.0(18.6-21.9)	0.771
Smoking, n(%)	1(6.25)	7(13.21)	0.527
Drinking, n(%)	0(0.00)	7(13.21)	0.210
Extrapulmonary tuberculosis, n(%)	5(31.25)	14(26.42)	0.704
**Baseline laboratory examination, median(IQR)**			
TBil umol/L	8.1(6.6-12.5)	9.2(7.0-12.1)	0.495
ALT IU/L	14.5(12.3-16.8)	14.0(10.0-20.5)	0.499
AST IU/L	21.5(15.3-26.0)	19.0(16.0-23.0)	0.339
Alkaline phosphatase, IU/L	74.5(57.8-89.0)	75.0(66.5-107.0)	0.518
Glutamyltranspeptidase, IU/L	18.0(11.8-34.3)	19.5(11.3-32.3)	0.750
Albumin, g/L	43.2(40.1-44.4)	44.9(42.2-46.8)	0.033
Creatinine, μmol/L	66.5(59.0-78.3)	67.0(58.5-76.0)	0.915
Uric acid, mmol/L	265.5(227.8-373.0)	314.0(265.5-370.0)	0.191
Hemoglobin, g/L	125.5(118.5-135.8)	137.0(128.5-145.5)	0.019
White blood cell ×10^12/L	6.8(5.4-8.5)	6.0(4.9-7.5)	0.060
Platelet×10^9/	242.5(182.0-417.3)	249.5(202.0-282.5)	0.937
ESR mm/h	20.0(10.8-83.3)	14.0(9.3-29.3)	0.098
C-reactive protein, mg/L	3.8(2.8-27.1)	3.4(2.1-8.6)	0.359
Triglyceride, mmol/L	1.1(0.7-1.5)	1.1(0.8-1.5)	0.889
**Circumstances of DILI, median (IQR)**			
Onset time, days	29.0(14.0-44.3)		
ALT, IU/L	168.0(129.0-331.0)		
AST, IU/L	155.0(122.0-362.0)		
TBil, umol/L	14.1(9.5-17.9)		
Alkaline phosphatase, IU/L	90.0(74.0-122.0)		

ALT, alanine aminotransferase; AST, aspartate aminotransferase; TBil, total bilirubin; ESR, erythrocyte sedimentation rate; BMI, body mass index; IQR, Interquartile distance.

After excluding patients with urinary system diseases, 14 ATB-DILI patients and 30 age- and sex- matched non-ATB-DILI patients were included for urinary metabolomics and microbiome analysis (**Supplementary Figure 1**). It is important to note that of these 14 patients, 8 met the definition of liver adaptation (45). The other 6 patients with ALT≥5 times the upper limit of normal were stopped using antituberculosis drugs according to Chinese guidelines, so it was hard to distinguish which were liver adaptation (36).

As shown in **Table 2**, there were no significant differences in sex, age, body weight, BMI, body mass index (BMI), smoking, drinking or tuberculosis site between the two groups of patients who underwent urine nontargeted metabolome and microbiome analysis (P>0.05). All participants had normal liver function before anti-tuberculosis drug ingestion. It was suggested that the general conditions of the two groups were consistent and comparable.

**Table 2:** Clinical characteristics of the two groups of matched patients.

Characteristic			Non-ATB-DILI group (n＝30)	ATB-DILI group (n＝14)	*P*	
Age, years, median(IQR)			33.0 (27.0-52.0)	43.5 (22.8-55.5)	0.772
Females, n(%)			16(53.3)	9(64.3)	0.495
Weight, kg, median(IQR)			52.8(48.0-58.8)	50.0(49.9-55.0)	0.495
BMI, kg/m2, median(IQR))			19.9(18.6-22.0)	19.5(18.7-20.1)	0.473
Smoking, n(%)			2(6.7)	1(7.1)	0.976
Drinking, n(%)			2(6.7)	0(0.0)	0.314
Extrapulmonary tuberculosis, n(%)			23(76.7)	11(78.6)	0.888

IQR, Interquartile distance.

### Metabonomic analysis of urine

**Figure 1:**
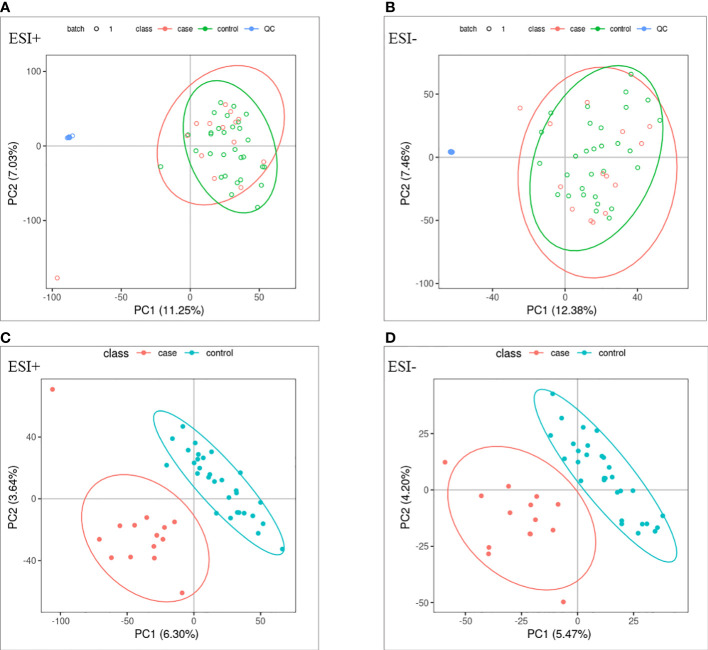
Multivariate statistical analysis. **(A, B)** principal component analysis scores scatter plots of the two groups. **(C, D)** partial least squares-discriminant analysis score scatter plots of the two groups. QC (blue circles), control group (n = 30, green circles), ATB-DILI group (n =14, red circles).

**Figure 2:**
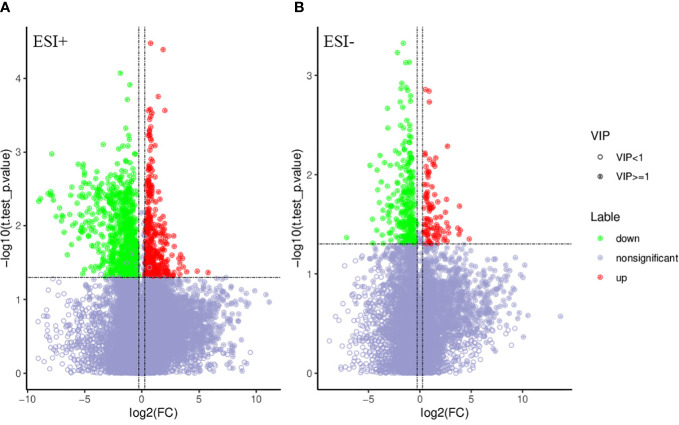
Volcano map of differential metabolites. Green is the down-regulated differential metabolite (labeled green), red is the up-regulated differential metabolite (labeled red), and metabolites without difference are labeled purple-gray. **(A)** positive ion mode, **(B)** negative ion mode.

PCA and OPLS-DA were performed for both positive ion mode (ESI+) and negative ion mode (ESI−). As shown in the figure (**Figures 1A, B**), the QC samples (blue circles) were significantly aggregated, indicating that the instrument was stable and that the reproducibility of the acquired data was good. The ATB-DILI (n=14, red circles) and tolerance groups (n=30, green circles) were not well separated in PCA. As shown (**Figures 1C, D**), the PLS-DA model clearly separated the ATB-DILI and non-ATB-DILI groups in both ionization modes. Differential metabolites between the two groups were screened according to multivariate and univariate statistical significance criteria (VIP≥1, FC≥1.2 or ≤ 0.83, and P<0.05). In general, there were 1256 urine differential metabolites screened in the positive ion mode and 334 in the negative ion mode (**Figure 2**). Finally, 28 differential metabolites with secondary classification names and reliable identification results (Level 1-3) were selected (**Table 3**), including choline, cherry base, N-acetyl, pseudohadine, N8-acetyl spermamine, glycolic acid, etc. As shown in **Table 4**, a total of 7 significant enrichment pathways for differential metabolites were found in both positive and negative ion modes. The differential metabolites were mainly involved in the metabolism of bile secretion, nicotinate and nicotinamide metabolism, tryptophan metabolism, ABC transporters, neuroactive ligand‒receptor interaction, arginine and proline metabolism, and porphyrin and chlorophyll metabolism (P<0.05, Count≥2) (**Table 4**).

**Table 3:** Identified differential metabolites between two groups.

Name	MW	RT	VIP	FC	P	Label
Choline	103.1	0.7	3.3	1.79	0.003	Up
Trigonelline	137.0	0.7	2.1	3.19	0.016	up
N-acetylputrescine	130.1	0.7	1.8	1.23	0.009	up
Pseudoephedrine	165.1	3.3	2.5	0.01	0.009	down
N8-acetylspermidine	187.2	0.7	2.4	1.43	0.004	up
Glycocholate	465.3	8.0	2.2	4.06	0.013	up
Uric acid	168.0	1.0	1.9	1.66	0.021	up
Ecgonine	185.1	4.9	1.8	1.96	0.028	up
1-methylnicotinamide	136.1	0.8	1.8	2.09	0.012	up
6-methylquinoline	143.1	3.5	1.6	0.63	0.035	down
Sebacic acid	202.1	6.4	1.6	1.97	0.049	up
Picolinic acid	123.0	3.5	1.5	0.64	0.012	down
3-hydroxyanthranilic acid	153.0	2.8	1.1	1.26	0.049	up
Mannitol	182.1	0.7	1.9	1.57	0.022	up
Carbendazim	191.1	0.7	1.6	0.15	0.023	down
Lipoamide	205.1	4.0	1.4	1.78	0.033	up
Ophthalmic acid	289.1	2.4	1.4	0.47	0.041	down
Valerophenone	162.1	5.5	1.2	0.71	0.030	down
D-(-)-lyxose	150.1	0.7	1.0	0.77	0.030	down
Creatine	131.1	0.7	1.6	0.32	0.030	down
L-glutamic acid	147.1	0.7	1.1	0.71	0.029	down
Methylmalonic acid	118.0	0.7	1.9	0.46	0.014	down
Porphobilinogen	226.1	0.7	1.5	0.49	0.001	down
Epinephrine	183.1	3.9	2.2	2.44	0.008	up
Heptanoic acid	130.1	5.4	1.4	0.50	0.047	down
11-dehydrothromboxane b2	368.2	6.8	1.1	1.40	0.040	up
Nonanoic acid	158.1	6.9	1.5	0.51	0.004	down
Taurolithocholic acid 3-sulfate	563.3	7.8	2.2	1.53	0.017	up

VIP, variable important for the projection; FC, fold-change; MW, molecular weight; RT, retention time.

**Table 4:** Differential metabolite pathway analysis.

Pathway	Ion modes	Count	Count All	P
Bile secretion	positive	4	97	<0.001
Nicotinate and nicotinamide metabolism	positive	2	55	0.001
Tryptophan metabolism	positive	2	81	0.002
ABC transporters	positive	2	124	0.005
Neuroactive ligand-receptor interaction	negative	2	52	<0.001
Arginine and proline metabolism	negative	2	78	<0.001
Porphyrin and chlorophyll metabolism	negative	2	142	0.003

### Correlation analysis of metabolic and clinical data

Correlation analysis was conducted between urine differential metabolites and clinical data, including baseline ALT, AST, TBIL, Alkaline phosphatase, hemoglobin, uric acid and albumin. We found that many different metabolites were significantly correlated with clinical data (**Supplementary Table 1**). The urine differential metabolite 11 dehydrothromboxane B2 was positively correlated with the baseline total bilirubin concentration, while the urine differential metabolite N8-acetylspermidine was negatively correlated with the hemoglobin content, and uric acid was also negatively correlated with the baseline serum uric acid level (**Supplementary Table 2**).

### Microbiome analysis of urine

**Figure 3:**
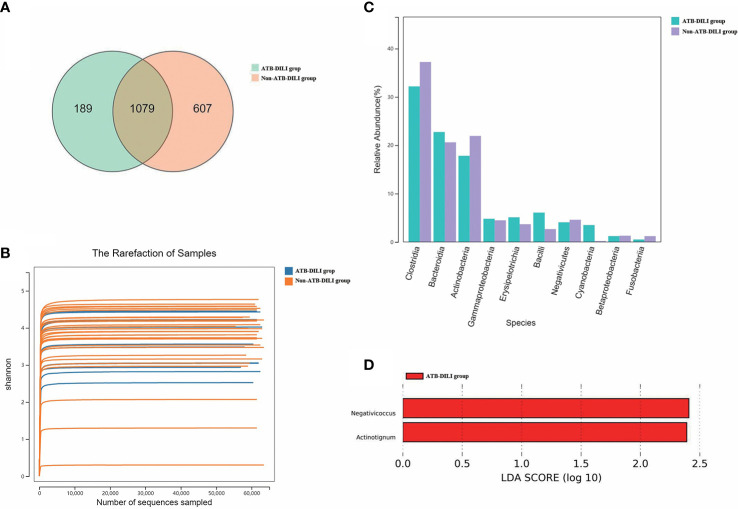
Comparison between 16S sequencing data of urine samples from non-ATB-DILI patients (n = 30) and ATB-DILI patients (n = 14). **(A)** Venn diagram. The left is the ATB-DILI group, the right is the control group. **(B)** α-diversity (Shannon curve). **(C)** Difference comparison of key species. **(D)** LEfSe analysis. Species with LDA greater than the set value of 2 are presented. The length of the bar indicates the magnitude of LDA influence.

As shown in the Figure (**Figure 3A**), 1079 OTUs were shared between the ATB-DILI group and the non-ATB-DILI group, 607 OTUs were unique to the non-ATB-DILI group, and the other 189 OTUs were unique to the ATB-DILI group. The Shannon curve (**Figure 3B**) shows that the amount of sequencing data in this study was large enough to reflect the vast majority of microbial information in the sample. The top 10 key species between the two groups are shown in **Figure 3C**. Weighted UniFrac principal coordinate analysis (PCoA) was applied to detect the changes in microbial community structures (**Supplementary Figure 3**). The results indicate that the ATB-DILI group and the control group were significantly separated along the PC2 axis, which explained 19.79% of the total variation. LEFSE analysis was used to determine the key attribute differences between the two groups. The differential microbiota (LDA score>2) screened between the two groups were Negativicoccus and Actinotignum, which were all upregulated in the ATB-DILI group (**Figure 3D**).

### Correlation of the urine microbiota and metabolism

**Figure 4:**
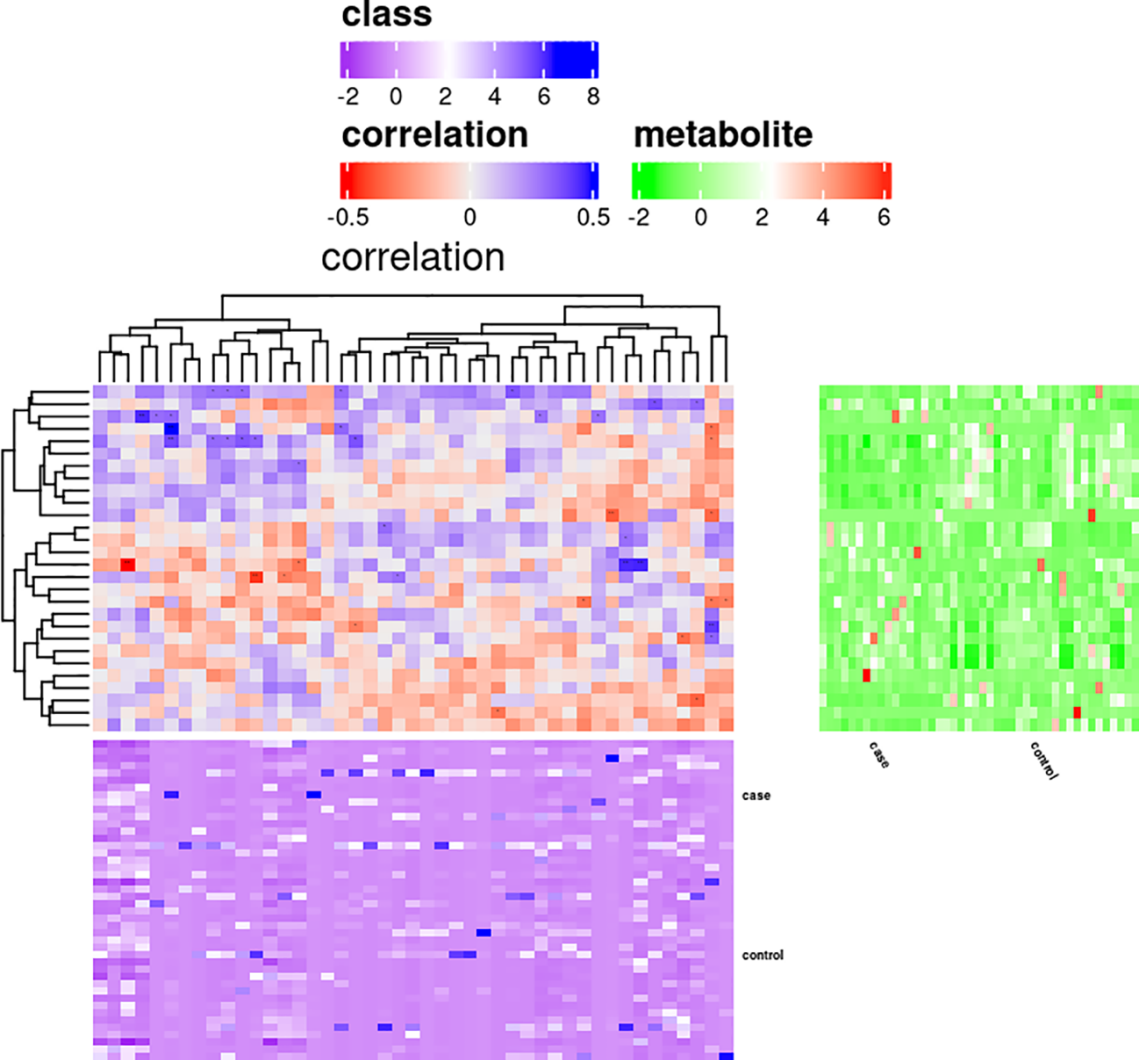
Correlation analysis of differential metabolites and microbiota.

We further investigated the correlation of urinary differential metabolites with altered urinary microbiota. Significant correlations were found between some differential metabolites and microbial groups by calculating rank correlation coefficients (**Figure 4** and **Supplementary Table 2**). Carbendazim was positively correlated with synergistia but negatively correlated with mollicutes (p<0.05) (**Supplementary Table 2**). D-(-)-lyxose was positively correlated with four microbial groups, including synergistia, ktedonobacteria, fibrobacteria and fusobacteriia (p<0.05) (**Supplementary Table 2**). Altogether, these results showed that distinctive metabolites were closely related to urinary microbiome variation, and distinctive metabolites and microbiomes were closely related to the occurrence of ATB-DILI.

### Subgroup analysis

According to RUCAM criteria, metabolome and microbiome analysis were performed between the ALT≥5 ULN group and normal patients. Significant metabolic differences were observed between the two groups, there were 1122 different metabolites were screened in positive ion mode and 386 in negative ion mode (**Supplementary Figure 3**). Finally, 26 different metabolites were selected, including choline, 11-dehydrothromboxane b2, and N8-acetylspermidine. (**Supplementary Table 3**). Consistent with our results in Section 3.2, 8 common different metabolites were found in the subgroup analysis to be related to liver injury after medication, especially when ALT>5ULN occurred (**Table 3** and **Supplementary Table 3**). The eight differential metabolites were choline, N8-acetylspermidine, carbendazim, N-acetylputrescine, 1-methylnicotinamide, creatine, porphobilinogen, and nonanoic acid (**Table 3** and **Supplementary Table 3**). And these different metabolites had the same label direction in the two groups of patients with liver injury (**Table 3** and **Supplementary Table 3**).

There were 795 OTUs shared between the DILI patients with ALT ≥ 5 ULN and normal patients, 68 OTUs were unique to the DILI group, and the other 891 OTUs were unique to the control group (**Supplementary Figure 4-A**). The top 10 key species between the two groups are shown in **Supplementary Figure 4-B**. Finally, 3 differential microbiotas (LDA score>2) were found between the two groups (**Supplementary Figure 4-B**). The Actinotignum was down regulated in DILI group, while the Bradyrhizobiaceae, and Bradyrhizobium were upregulated in the non-DILI group (**Supplementary Figures 4-C**). Combined with the results in Section 3.4, we have sufficient evidence to show that Actinotignum was closely related to the occurrence of liver injury after medication, regardless of the DILI standards.

### Comparison of the models for the prediction of ATB-DILI

**Figure 5:**
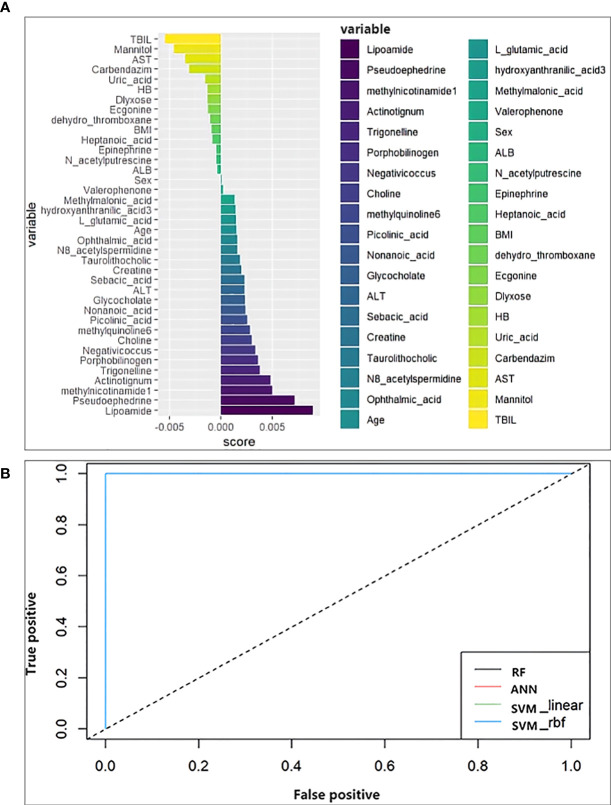
Machine learning models. **(A)** Score the importance of variables. The larger the value, the more important the variable is. **(B)** Receiver operating characteristic curve for the models developed with the top 10 important variables as inputs. ALT, alanine aminotransferase; AST, aspartate aminotransferase; TBIL, total bilirubin; ALB, albumin; BMI, body mass index; HB, hemoglobin.

Random forest analysis was performed on the screened differential metabolites (**Table 3**), differential microbiota (**Figure 2**), and relevant clinical data of 44 patients. For clinical characteristics, we included albumin and hemoglobin, which were significantly different between the two groups, as well as other factors that may be associated with the occurrence of ATB-DILI (including age, sex, BMI, baseline ALT, AST, and TBil). A total of 38 variables are included. When ntree=500 and mtry=6, the model reaches the optimum. The score of the 38 variables was shown in **Figure 5A**. The larger the absolute value is, the greater the importance of the indicator. After sorting the variables from high to low according to the absolute value, the cross-validation curve was obtained by performing tenfold cross-validation repeated 5 times (**Supplementary Figure 5**). The top 10 variables were selected for model building with the lowest error (**Supplementary Figure 5**). The area under the ROC curve of the four models were shown in **Table 5**. At training set, the random forest model performed significantly better than the remaining three models (area under the curve 0.98 vs. 0.87 (ANN), 0.89 (SVM-linear) and 0.89 (SVM-rbf) (**Table 5**). Overall, random forest model, artificial neural network model and two support vector machine models (both SVM-linear and SVM-rbf) all have excellent prediction value for the validation set (**Figure 5B** and **Table 5**). The consistent results between the training set and the validation set indicate that those models have high accuracy for predicting the occurrence of ATB-DILI.

**Table 5:** The area under the curve of machine learning models.

Model	AUC	
	Training set	Test set
RF	0.98	1
ANN	0.87	1
SVM_linear	0.89	1
SVM_rbf	0.89	1

AUC, area under the curve; RF, random forest; ANN, artificial neural network; SVM, support vector machine; SVM_rbf, support vector machine with radial basis function kernel.

## Discussion

Evidence that the human urine microbiome and metabolome contribute to the development of ATB-DILI is accumulating. Thus, characterization of the urinary microbiota and metabolites in ATB-DILI is highly warranted, especially before medication. Herein, we first reported the characterization of urine metabolomics and the microbiome in patients with ATB-DILI before medication and identified key metabolites and bacteria that may be involved in the development of ATB-DILI. Meanwhile, we first proposed and successfully built four ATB-DILI clinical prediction models using our metabolomics, microbiome and clinical data.

In this study, the levels of ALT, AST, TBil or ALP were within the normal range in all enrolled patients before ingestion of anti-tuberculosis drugs. Approximately 23.2% of the patients had markedly elevated ALT and AST after ingesting anti-tuberculosis drugs. According to China’s 2019 guidelines for the diagnosis and treatment of ATB-DILI (36), for DILI caused by anti-tuberculosis drugs, when ALT≥3ULN or TBil≥2ULN, the relevant anti-tuberculosis drugs need to be discontinued, and when ALT≥5ULN or TBil≥3ULN, it is necessary to stop all anti-tuberculosis drugs. Indicates that DILI needs to be taken seriously in TB patients. Therefore, we first analyzed the characteristics of the metabolomics and microbiome of DILI patients with ALT≥3 ULN. As a large number of domestic and foreign studies both recommend the use of RUCAM to assess DIL (46–48), we did a subgroup analysis for those DILI was defined as serum ALT level ≥5ULN. What was exciting was that no matter which DILI standard, we have found the same differential metabolites and microorganisms.

Metabolomics and the microbiome were used to analyze the urine of the ATB-DILI susceptible group and normal liver function control group, and the two groups could be distinguished significantly on the PLS-DA scatter plot. Consistent with those of a previous study (23, 24, 26), our results also indicated that ATB-DILI susceptible individuals may have specific metabolomic and microbiological patterns. We identified 28 major differential metabolites between the two groups in urine, including choline, trigonelline, N-acetylputrescine, uric acid, etc. The biological properties of each metabolite were searched from the human metabolome database (https://hmdb.ca/), and summarized in **Supplementary Table 4**. The differential metabolites selected in this study were consistent with the biospecimen locations in the database. This process involves bile secretion, nicotinate and nicotinamide metabolism, tryptophan, ABC transporters, neuroactive ligand-receptor interaction, arginine and proline metabolism, porphyrin and chlorophyll metabolism. Two major differential microbial, Negativicoccus and Actinotignum, were identified between the two groups.

As an essential nutrient, choline in the urine of patients with overactive bladder was 34.8% lower in urine metabolomic analysis than patients without overactive bladder (P = 0.014) (49). The urinary excretion of choline metabolites in term breast-fed infants was significantly higher than that in term formula-fed infants (P < 0.05) (50). This study found that urinary choline levels may be a noninvasive biomarker for predicting ATB-DILI. For the first time, upregulated trigonelline in urine before medication was found to be associated with ATB-DILI in TB patients. This may be related to the possible inhibition of key enzymes in lipid metabolism and absorption by trigonelline (51). Previous studies have found that glycocholic acid levels were significantly increased in DILI (52, 53), and the increased levels were positively correlated with the severity of DILI (52). Combined with the results of this study, glycocholic acid may have a role as a biomarker for DILI. As a proinflammatory and proapoptotic molecule, uric acid plays an intermediary role in the process of liver and kidney injury (54, 55), and animal experiments have shown that the elevation of uric acid may lead to alcohol-induced steatosis, endoplasmic reticulum stress, and cell apoptosis. death and liver damage (56). Cao et al. found that uric acid levels in urine can be used to differentiate ATB-DILI from non-ATB-DILI patients (24). In this study, we found that uric acid in urine generation before medication could be used as a biomarker to predict the occurrence of ATB-DILI after medication, indicating that uric acid in urine metabolism may have great potential in predicting and identifying ATLI.

Additionally, differential metabolite enrichment analysis showed that metabolic pathways, including bile secretion, niacin and nicotinamide metabolism, ABC transporters, and etc., were involved in the occurrence of ATB-DILI after medication. Impaired bile secretion has been observed in mouse models of liver injury (57), which leads to intrahepatic bile accumulation (58). Studies have found that the bile secretion pathway is involved in psoralen (59) and liver injury induced by baklavaine (60). Our study found that abnormalities in the bile secretion pathway existed before liver injury occurred and before drug administration. It has also been shown that liver damage can be alleviated by improving bile secretion (61). Therefore, this pathway may provide a new target for the prevention and treatment of ATB-DILI. Consistent with our earlier study, niacin and nicotinamide metabolism were involved in the occurrence of ATB-DILI in this study (26). The difference is that a previous study found that the niacin and nicotinamide metabolism pathways were significantly altered when liver injury occurred, and this study found that this pathway abnormality existed long before liver injury occurred. Herein, the niacin and nicotinamide metabolic pathways play an important role in the development and progression of ATLI. The specific mechanism needs further study.

Each disease has its own unique microbial alterations (29, 62). Microorganisms in the gut originate from the digestive system, while urine microorganisms reflect the entire body including the intestinal tract, oral system, respiratory system, etc (63). Studies have shown that the urinary microbiota is associated with diseases outside the urinary system (64, 65). Previous studies have indicated that microbiota alterations are associated with drug-induced liver injury (26, 32, 33). Our previous study found that six microbiota including o_Bacteroidales, f_Prevotellaceae, etc., were associated with ATB-DILI (26). Compared with control group, this prospective study found that the Negativicoccus and Actinotignum were upregulated in the ATB-DILI group before medication. Negativicoccus was found to be significantly increased in the oral cavity of hamsters using smokeless tobacco products (66). Among patients with nonmuscle-invasive bladder cancer, BCG-vaccinated patients had significantly more negativicoccus in their urine than nonvaccinated BCG patients (67). Negativicoccus was also found to be one of the core flora in all ground glass nodules and normal tissue samples (68). However, studies have proven that Negativicoccus and Actinotignum are associated with ATB-DILI.

Furthermore, our results also suggested that there may be specific metabolomic and microbiological patterns in individuals susceptible to severe ATB-DILI when compared with the mild ATB-DILI group. The discovery of these biomarkers may help with the early identification of TB patients at risk of developing severe DILI, thus providing new ideas for the individualized treatment of TB. However, due to the limited sample size, the results of this study cannot be directly generalized to other populations.

This study is the first to establish the early prediction models of ATB-DILI by combining clinical data and metabolomics and microbiology data using a machine learning method. The random forest algorithm was used to analyze multiple variables, the importance of each variable was scored, and the optimal variable (top 10) combination was obtained by adjusting the parameters to form the ATB-DILI prediction models. The results of the training set and the validation set were consistent (all ROC ≥ 0.85) (**Table 5**). Based on clinical and genomic data, researchers from Taipei Medical University compared the accuracy of multiple machine learning methods in predicting ATB-DILI, among which the artificial neural network showed the best prediction performance (69). In their study, the area under the ROC curve of the training set in the random forest algorithm was 0.724 and 0.718 for the validation set (69). Combined with our study, machine learning techniques show great potential in predicting ATB-DILI and may provide new opportunities for the diagnosis and treatment of ATB-DILI.

This study has some limitations. First, the number of participants was limited. However, this was a prospective study, which enhanced reliability of the results. Further validation in more centers with more patients needs to be verified in the future. Second, even though this study adds to the understanding of metabolome and microbiological patterns on the progress of ATD-DILI, this study only analyzed predose characteristics and lacked data at multiple time points after drug use. There is much work yet to be performed to understand these changes entirely. Finally, the current study obtained good predictive value in both the training set and the validation set, but limited by the limited sample size and geographical limitations, further verification is required in studies with more regions and larger samples in the future.

## Conclusion

In conclusion, our findings extend our knowledge of the relationship between urinary metabolites and microbiota and host ATB-DILI susceptibility, indicating that certain metabolomic and microbiome changes from the host can be used to identify and predict an individual’s susceptibility to ATB-DILI. In the future, prospective cohorts with a larger number of subjects are needed to investigate the potential clinical utility of metabolic markers in the identification of susceptible individuals. Prospective cohorts with more subjects and more time points are needed to investigate the potential clinical utility of metabolic markers and key microbiota in identifying susceptible individuals.

## Data availability statement

The original contributions presented in the study are publicly available. This data can be found here: https://www.ncbi.nlm.nih.gov/, PRJNA870240.

## Ethics statement

The studies involving human participants were reviewed and approved by West China Hospital of Sichuan University [Approval No.: 761 (2019)]. The patients/participants provided their written informed consent to participate in this study.

## Author contributions

All authors contributed substantially to the study design, data interpretation, and the writing of the manuscript. J-QH contributed to the study design. M-GW and S-QW contributed to data collection and analysis and completed the full text. M-MZ contributed to data collection. All authors contributed to the article and approved the submitted version.

## Funding

This work was supported by the National Natural Science Foundation of China (Grant No. 81870015).

## Conflict of interest

The authors declare that the research was conducted in the absence of any commercial or financial relationships that could be construed as a potential conflict of interest.

## Publisher’s note

All claims expressed in this article are solely those of the authors and do not necessarily represent those of their affiliated organizations, or those of the publisher, the editors and the reviewers. Any product that may be evaluated in this article, or claim that may be made by its manufacturer, is not guaranteed or endorsed by the publisher.

## Supplementary material

Supplementary Figure 1 Flow chart.

Supplementary Figure 2 Volcano map of differential metabolites in subgroup analysis.

Supplementary Figure 3 Principal co-ordinates analysis.

Supplementary Figure 4 Subgroup analysis of 16S sequencing data of urine samples. **(A)** Venn diagram. The left is the severe DILI group, the right is the non-DILI group. **(B)** Difference comparison of the top 10 key species. **(C)** LEfSe analysis. Species with LDA greater than the set value of 2 are presented. The length of the bar indicates the magnitude of LDA influence.

Supplementary Figure 5 Cross validation curve. The abscissa is the number of variables, and the ordinate is the cross-validation error rate.
