# Supplementary figures and images for: Urine metabolomics and microbiome analyses reveal the mechanism of anti-tuberculosis drug-induced liver injury, as assessed for causality using the updated RUCAM: A prospective study
Source: Front Immunol. 2022 Nov 22;13:1002126. doi: 10.3389/fimmu.2022.1002126 (PMC9724621; doi:10.3389/fimmu.2022.1002126)

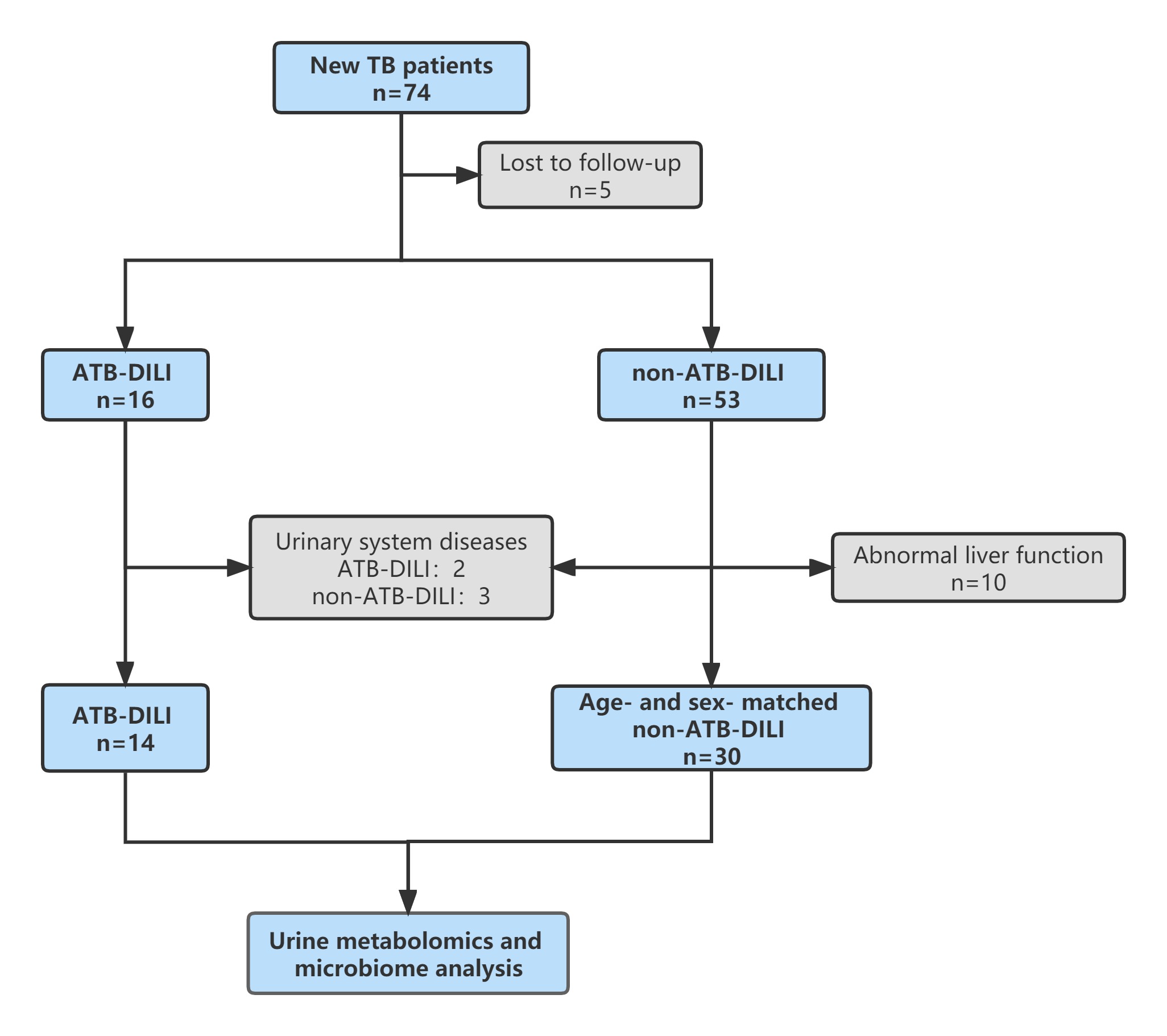

Supplement: Supplementary Figure 1 — Flow chart. [file Image_1.jpeg]

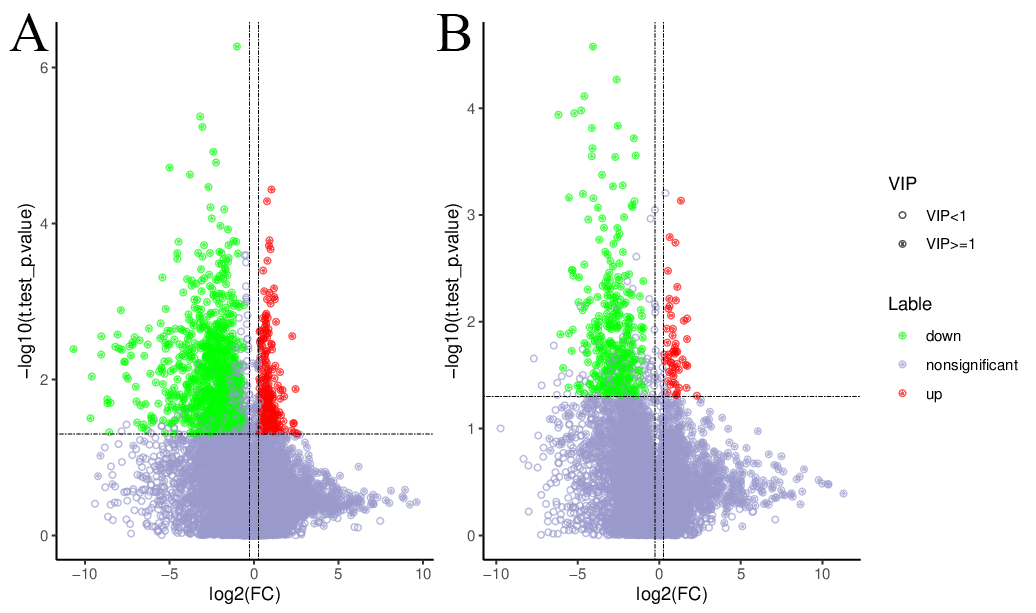

Supplement: Supplementary Figure 2 — Volcano map of differential metabolites in subgroup analysis. [file Image_2.tif]

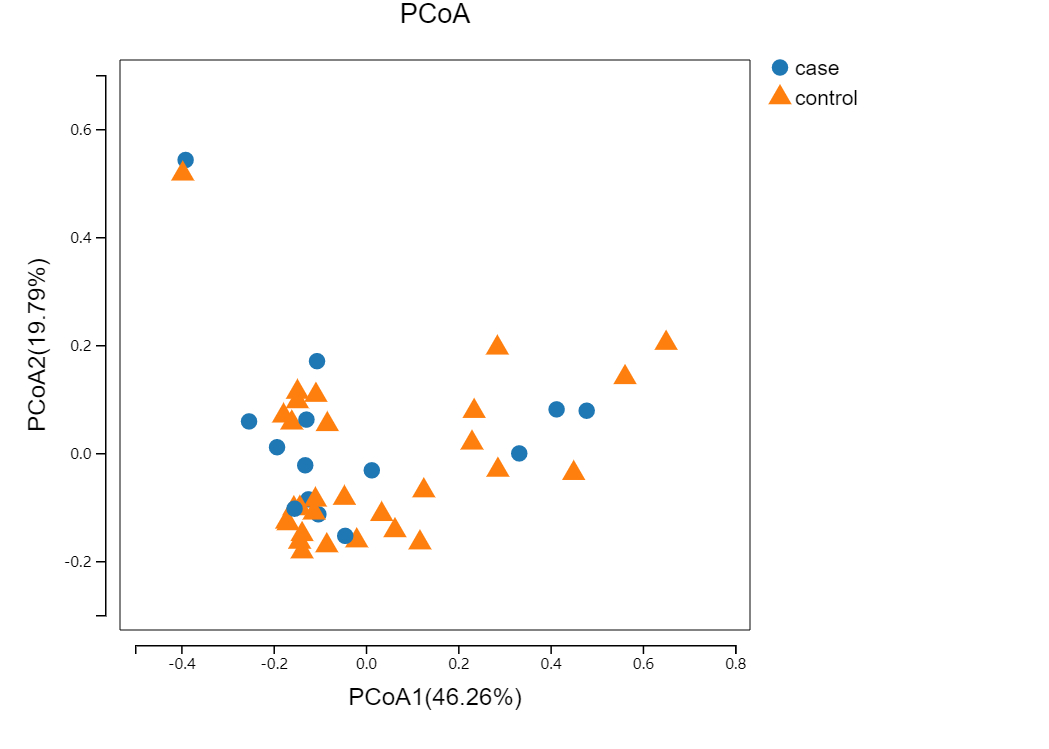

Supplement: Supplementary Figure 3 — Principal co-ordinates analysis. [file Image_3.jpeg]

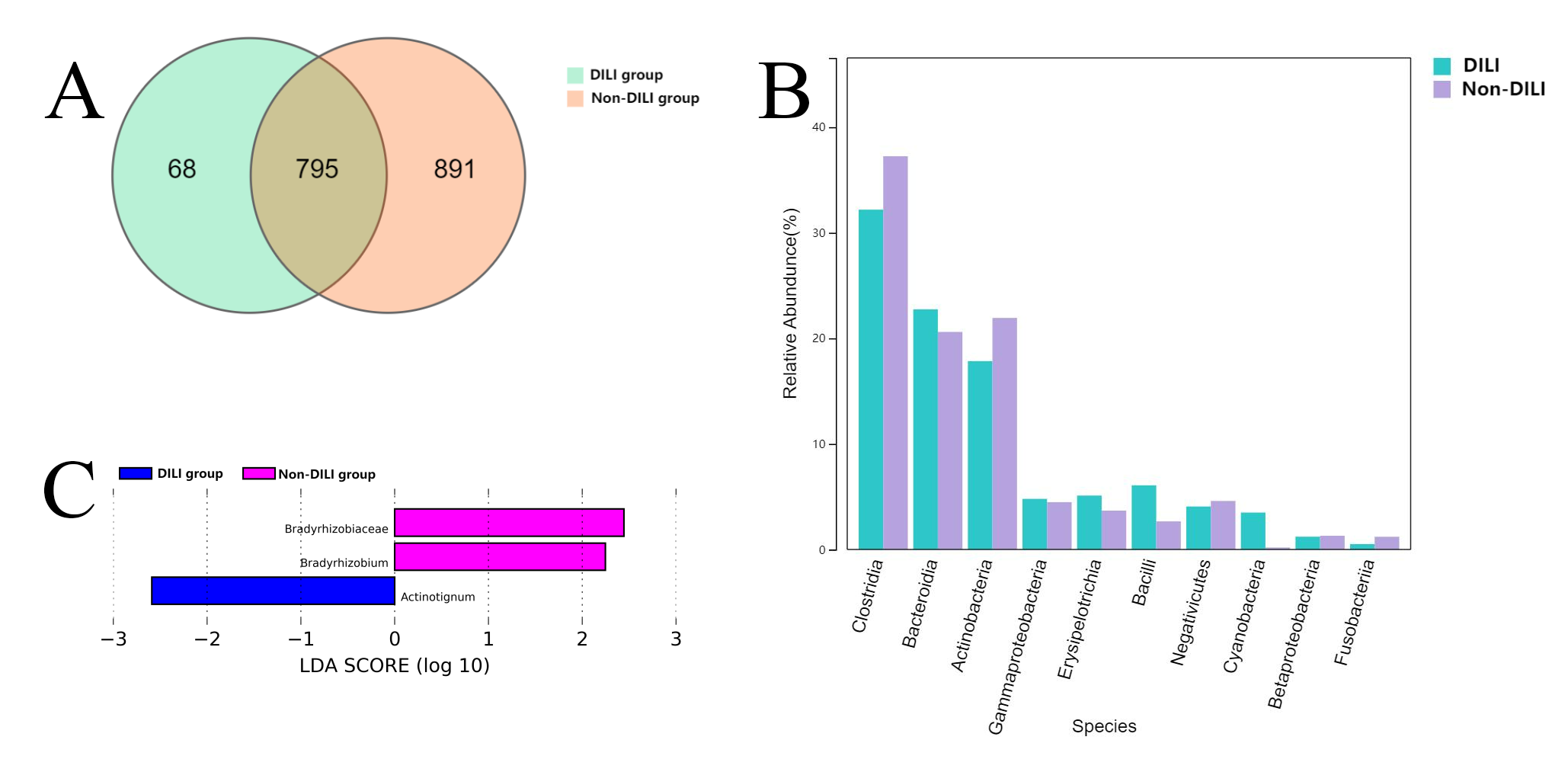

Supplement: Supplementary Figure 4 — Subgroup analysis of 16S sequencing data of urine samples. (A) Venn diagram. The left is the severe DILI group, the right is the non-DILI group. (B) Difference comparison of the top 10 key species. (C) LEfSe analysis. Species with LDA greater than the set value of 2 are presented. The length of the bar indicates the magnitude of LDA influence. [file Image_4.tif]

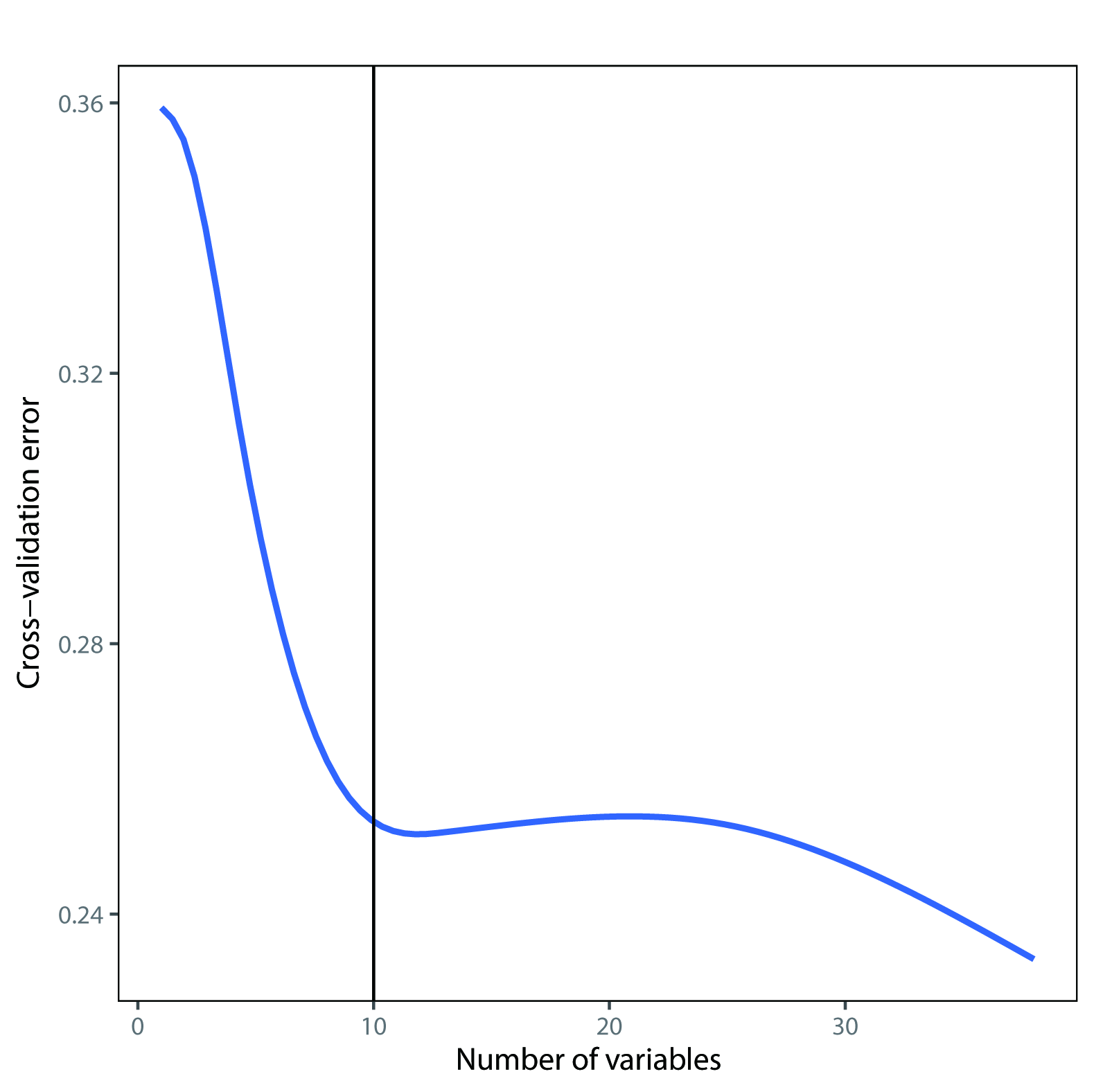

Supplement: Supplementary Figure 5 — Cross validation curve. The abscissa is the number of variables, and the ordinate is the cross-validation error rate. [file Image_5.tiff]
