# Supplementary material for: Urine metabolomics and microbiome analyses reveal the mechanism of anti-tuberculosis drug-induced liver injury, as assessed for causality using the updated RUCAM: A prospective study
Source: Front Immunol. 2022 Nov 22;13:1002126. doi: 10.3389/fimmu.2022.1002126 (PMC9724621; doi:10.3389/fimmu.2022.1002126)
Supplement: Supplementary file 6 [file Table_1.docx]

**Supplementary table**

Supplementary Table 1 Correlation of the urine metabolic and clinical data

| **Metabolites** | **Clinical parameters** | **R** | **P** |
| --- | --- | --- | --- |
| 1-methylnicotinamide | Uric acid | -0.3 | 0.048 |
| 11-dehydrothromboxane b2 | TBil | 0.5 | <0.001 |
| 3-hydroxyanthranilic acid | Albumin | -0.4 | 0.014 |
| 3-hydroxyanthranilic acid | Alkaline phosphatase | -0.3 | 0.030 |
| 3-hydroxyanthranilic acid | White blood cell | 0.4 | 0.003 |
| 6-methylquinoline | Albumin | 0.4 | 0.017 |
| Carbendazim | Alkaline phosphatase | -0.3 | 0.027 |
| Carbendazim | Glutamyltranspeptidase | -0.4 | 0.002 |
| Choline | Albumin | -0.3 | 0.030 |
| Choline | AST | 0.4 | 0.016 |
| Choline | CreatinineA | -0.3 | 0.044 |
| Choline | Uric acid | -0.3 | 0.025 |
| Ecgonine | Platelet | -0.3 | 0.021 |
| Epinephrine | Platelet | -0.4 | 0.018 |
| Glycocholate | Albumin | -0.4 | 0.002 |
| Glycocholate | Glutamyltranspeptidase | 0.3 | 0.021 |
| Glycocholate | Platelet | 0.4 | 0.013 |
| Glycocholate | White blood cell | 0.3 | 0.029 |
| Heptanoic acid | CreatinineA | 0.3 | 0.048 |
| Heptanoic acid | Uric acid | 0.3 | 0.021 |
| Lipoamide | Albumin | -0.4 | 0.016 |
| Mannitol | Glutamyltranspeptidase | 0.4 | 0.015 |
| Mannitol | Uric acid | -0.3 | 0.050 |
| N-acetylputrescine | Albumin | -0.3 | 0.032 |
| N-acetylputrescine | AST | 0.4 | 0.005 |
| N-acetylputrescine | CreatinineA | -0.4 | 0.013 |
| N8-acetylspermidine | Albumin | -0.5 | <0.001 |
| Porphobilinogen | Uric acid | 0.5 | 0.001 |
| Sebacic acid | Uric acid | -0.4 | 0.002 |
| Taurolithocholic acid 3-sulfate | C-reactive protein | 0.3 | 0.039 |
| Taurolithocholic acid 3-sulfate | Uric acid | -0.3 | 0.031 |
| Trigonelline | CreatinineA | -0.4 | 0.012 |
| Trigonelline | Platelet | -0.3 | 0.049 |
| Uric acid | Uric acid | -0.6 | <0.001 |
| Valerophenone | Albumin | 0.4 | 0.006 |

Abbreviation: AST, aspartate aminotransferase; TBil, total bilirubin.

Supplementary Table 2 Correlation of the urine metabolic and microbiota

| **Metabolites** | **Microbiota** | **R** | **P** |
| --- | --- | --- | --- |
| Carbendazim | Synergistia | 0.5 | 0.001 |
| Taurolithocholic acid 3-sulfate | Clostridia | -0.5 | 0.001 |
| Taurolithocholic acid 3-sulfate | Actinobacteria | 0.5 | 0.002 |
| Taurolithocholic acid 3-sulfate | Thermomicrobia | 0.5 | 0.002 |
| Lipoamide | Negativicutes | 0.5 | 0.002 |
| 11-dehydrothromboxane b2 | Fusobacteriia | -0.4 | 0.002 |
| Choline | Mollicutes | 0.4 | 0.004 |
| D-(-)-lyxose | Synergistia | 0.4 | 0.009 |
| Pseudoephedrine | Cyanobacteria | -0.4 | 0.010 |
| Uric acid | Spartobacteria | 0.4 | 0.013 |
| Methylmalonic acid | Flavobacteriia | -0.4 | 0.013 |
| D-(-)-lyxose | Ktedonobacteria | 0.4 | 0.014 |
| Nonanoic acid | Gemmatimonadetes | 0.4 | 0.015 |
| Pseudoephedrine | Mollicutes | -0.4 | 0.017 |
| D-(-)-lyxose | Fibrobacteria | 0.4 | 0.018 |
| 1-methylnicotinamide | Gammaproteobacteria | -0.3 | 0.021 |
| Sebacic acid | Acidobacteria_Gp3 | -0.3 | 0.026 |
| Carbendazim | Mollicutes | -0.3 | 0.027 |
| D-(-)-lyxose | Fusobacteriia | 0.3 | 0.028 |
| Creatine | Acidobacteria_Gp7 | -0.3 | 0.029 |

Supplementary Table 3 Identified differential metabolites in the subgroup analysis.

| **Name** | **MW** | **RT** | **VIP** | **FC** | **P** | **label** |
| --- | --- | --- | --- | --- | --- | --- |
| L-lysine | 146.1 | 0.6 | 1.3 | 0.49 | 0.043 | down |
| Pipecolate | 129.1 | 0.6 | 1.3 | 0.49 | 0.043 | down |
| Choline | 103.1 | 0.7 | 3.2 | 2.06 | 0.004 | up |
| N8-acetylspermidine | 187.2 | 0.7 | 2.5 | 1.48 | 0.003 | up |
| Carbendazim | 191.1 | 0.7 | 1.6 | 0.15 | 0.035 | down |
| N-acetylputrescine | 130.1 | 0.7 | 2.0 | 1.28 | 0.011 | up |
| 1-methylnicotinamide | 136.1 | 0.8 | 1.5 | 1.60 | 0.016 | up |
| Formyl-l-methionyl peptide | 177.0 | 2.8 | 1.7 | 1.70 | < 0.001 | up |
| Pyridoxine | 169.1 | 3.1 | 1.8 | 0.27 | 0.015 | down |
| 5,6-dimethylbenzimidazole | 146.1 | 3.1 | 2.5 | 2.47 | 0.002 | up |
| Sinomenine | 329.2 | 3.4 | 1.1 | 0.03 | 0.048 | down |
| Suberic acid | 174.1 | 3.5 | 1.0 | 0.70 | 0.038 | down |
| 4-(2-aminopropyl)-2-methoxyphenol | 181.1 | 3.7 | 1.1 | 1.37 | 0.017 | up |
| Isoliquiritigenin | 256.1 | 4.8 | 2.2 | 0.05 | 0.031 | down |
| 5-methoxysalicylic acid | 168.0 | 5.3 | 2.2 | 0.23 | 0.024 | down |
| Trans-cinnamaldehyde | 132.1 | 5.7 | 1.1 | 1.32 | 0.043 | up |
| Estriol | 288.2 | 6.4 | 1.0 | 0.01 | 0.019 | down |
| Capsaicin | 305.2 | 7.3 | 1.8 | 0.15 | 0.002 | down |
| Creatine | 131.1 | 0.7 | 1.4 | 0.21 | 0.002 | down |
| Porphobilinogen | 226.1 | 0.7 | 1.2 | 0.48 | 0.009 | down |
| Benzoic acid | 122.0 | 1.3 | 1.7 | 1.74 | 0.030 | up |
| D-(-)-salicin | 286.1 | 3.5 | 1.5 | 0.12 | 0.024 | down |
| 4-phenylbutyric acid | 164.1 | 6.1 | 1.1 | 0.03 | 0.045 | down |
| Geranylgeranyl pyrophosphate | 450.2 | 6.7 | 1.3 | 0.04 | 0.016 | down |
| 11-dehydro thromboxane b2 | 368.2 | 6.8 | 1.6 | 2.04 | 0.019 | up |
| Nonanoic acid | 158.1 | 6.9 | 1.9 | 0.35 | 0.001 | down |

Abbreviation: VIP, variable important for the projection; FC, fold-change; MW, molecular weight; RT, retention time.

Supplementary Table 4 The biological properties the differential metabolites.

| **Metabolites** | **Cellular Locations** | **Biospecimen Locations** | **Tissue Locations** | **Pathways** |
| --- | --- | --- | --- | --- |
| Choline | Cytoplasm  Extracellular  Mitochondria  Nucleus  Endoplasmic reticulum  Golgi apparatus | Blood  Breast Milk  Cerebrospinal Fluid (CSF)  Feces  Saliva  Sweat  Urine | Basal Ganglia  Bladder  Brain  Epidermis  Fibroblasts  Intestine  Kidney  Liver  Neuron  Placenta  Platelet  Prostate  Skeletal Muscle  Spleen  Testis | Glycine, serine and threonine metabolism;Methionine Metabolism; Phospholipid Biosynthesis; Cystathionine Beta-Synthase Deficiency; Hypermethioninemia, et al. |
| Trigonelline | Cytoplasm | Blood  Feces  Urine | Placenta | Not Available |
| N-acetylputrescine | Cytoplasm (predicted from logP) | Blood  Feces  Saliva  Urine | Placenta | Not Available |
| Pseudoephedrine | Not Available | Blood  Feces  Urine | Not Available | Not Available |
| N8-acetylspermidine | Not Available | Blood  Saliva  Urine | Not Available | Not Available |
| Glycocholate | Extracellular | Blood  Feces  Urine | Fibroblasts  Liver  Placenta  Prostate | Primary bile acid biosynthesis; Congenital Bile Acid Synthesis Defect Type II; Familial Hypercholanemia; Zellweger Syndrome, et al. |
| Uric acid | Extracellular  Peroxisome | Amniotic Fluid  Bile  Blood  Cerebrospinal Fluid (CSF)  Feces  Saliva  Urine | Adipose Tissue  Bladder  Brain  Epidermis  Erythrocyte  Intestine  Kidney  Liver  Placenta  Platelet  Prostate  Skeletal Muscle  Spleen | Purine metabolism; Adenosine Deaminase Deficiency; Adenylosuccinate Lyase Deficiency; Gout or Kelley-Seegmiller Syndrome, et al. |
| Ecgonine | Not Available | Not Available | Not Available | Not Available |
| 1-methylnicotinamide | Cytoplasm  Extracellular | Blood  Feces  Urine | Placenta | Nicotinate and nicotinamide metabolism |
| 6-methylquinoline | Membrane | Not Available | Not Available | Not Available |
| Sebacic acid | Membrane (predicted from logP) | Blood  Feces  Urine | Not Available | Not Available |
| Picolinic acid | Cytoplasm  Extracellular | Amniotic Fluid  Blood  Cerebrospinal Fluid (CSF)  Feces  Saliva  Urine | Intestine  Liver  Placenta  Prostate | Not Available |
| 3-hydroxyanthranilic acid | Cytoplasm  Membrane | Blood  Urine | Bladder  Epidermis  Leukocyte | Tryptophan metabolism |
| Mannitol | Extracellular  Membrane | Blood  Cerebrospinal Fluid (CSF)  Feces  Saliva  Urine | Brain  Epidermis  Placenta | Not Available |
| Carbendazim | Cytoplasm  Extracellular | Not Available | Not Available | Not Available |
| Lipoamide | Membrane (predicted from logP)  Mitochondria | Saliva | Placenta | Valine, leucine and isoleucine degradation;Citric Acid Cycle; Congenital lactic acidosis; Fumarase deficiency; Mitochondrial complex II deficiency, et al. |
| Ophthalmic acid | Not Available | Not Available | Placenta | Not Available |
| Valerophenone | Membrane | Not Available | Not Available | Not Available |
| D-(-)-lyxose | Extracellular  Lysosome | Urine | Adipose Tissue  Bladder  Epidermis  Fibroblasts  Intestine  Kidney  Neuron  Placenta  Platelet  Prostate  Skeletal Muscle  Spleen  Testis | Not Available |
| Creatine | Cytoplasm  Extracellular  Mitochondria | Blood  Breast Milk  Cerebrospinal Fluid (CSF)  Feces  Saliva  Sweat  Urine | Adipose Tissue  Bladder  Brain  Epidermis  Fibroblasts  Heart  Intestine  Kidney  Neuron  Placenta  Platelet  Prostate  Skeletal Muscle  Spleen  Testis | Glycine, serine and threonine metabolism; Arginine and proline metabolism; Prolidase Deficiency; Hyperprolinemia Type II, et al. |
| L-glutamic acid | Extracellular  Mitochondria  Lysosome  Endoplasmic reticulum | Blood  Cellular Cytoplasm  Cerebrospinal Fluid (CSF)  Feces  Saliva  Sweat  Urine | Adipose Tissue  Adrenal Medulla  Epidermis  Fibroblasts  Intestine  Kidney  Neuron  Pancreas  Placenta  Platelet  Prostate  Skeletal Muscle  Spleen | Alanine, aspartate and glutamate metabolism; Histidine metabolism; Malate-Aspartate Shuttle; Nitrogen metabolism, et al. |
| Methylmalonic acid | Cytoplasm (predicted from logP) | Blood  Cerebrospinal Fluid (CSF)  Feces  Urine | Kidney  Liver  Placenta | Valine, leucine and isoleucine degradation; Propanoate metabolism; Beta-Ketothiolase Deficiency; 2-Methyl-3-Hydroxybutryl CoA Dehydrogenase Deficiency, et al. |
| Porphobilinogen | Cytoplasm (predicted from logP) | Blood  Urine | Erythrocyte  Liver | Porphyrin Metabolism; Acute Intermittent Porphyria; Porphyria Variegata; Hereditary Coproporphyria, et al. |
| Heptanoic acid | Extracellular  Membrane (predicted from logP) | Feces  Saliva | Kidney | Not Available |
| 11-dehydrothromboxane b2 | Extracellular  Membrane (predicted from logP) | Blood  Urine | Platelet | Arachidonic Acid Metabolism；Leukotriene C4 Synthesis Deficiency；Piroxicam Action Pathway；Acetylsalicylic Acid Action Pathway；Etodolac Action Pathway， et al. |
| Taurolithocholic acid 3-sulfate | Extracellular | Blood  Feces  Urine | Not Available | Not Available |
| Nonanoic acid | Extracellular  Membrane | Blood  Feces  Saliva  Sweat  Urine | Epidermis | Not Available |
| Epinephrine | Cytoplasm  Extracellular | Blood  Cerebrospinal Fluid (CSF)  Urine | Adipose Tissue  Adrenal Cortex  Adrenal Gland  Adrenal Medulla  Bladder  Epidermis  Eye Lens  Fibroblasts  Intestine  Kidney  Liver  Neuron  Ovary  Pancreas  Placenta  Platelet  Skeletal Muscle  Testis | Tyrosine metabolism;Aromatic L-Aminoacid Decarboxylase Deficiency;Tyrosine hydroxylase deficiency;Alkaptonuria, et al. |
